# Use of the Internet in Scanning the Horizon for New and Emerging Health Technologies: A Survey of Agencies Involved in Horizon Scanning

**DOI:** 10.2196/jmir.5.1.e6

**Published:** 2003-03-31

**Authors:** Karla Douw, Hindrik Vondeling, Drea Eskildsen, Sue Simpson

**Affiliations:** ^1^University of Southern DenmarkDepartment of Health EconomicsInstitute of Public HealthOdenseDenmark; ^2^Danish Centre for Evaluation and Health Technology AssessmentCopenhagenDenmark; ^3^University of BirminghamNational Horizon Scanning CentreDepartment of Public Health & EpidemiologyBirminghamUK

**Keywords:** Horizon scanning, Health Technology, Technology Assessment, Biomedical, Internet, Survey

## Abstract

**Background:**

A number of countries worldwide have structured horizon scanning systems which provide timely information on the impact of new health technologies to decision makers in health care. In general, the agencies that are responsible for horizon scanning have limited resources in terms of budget and staff. In contrast, the number of new and emerging health technologies, i.e. pharmaceuticals, medical devices, and medical and surgical procedures, is growing rapidly. This requires the Horizon Scanning Systems (HSSs) to devise efficient procedures for identification of new health technologies. The role of the Internet for this purpose has as yet not been documented.

**Objective:**

To describe and analyse how the Internet is used by horizon scanning systems to systematically identify new health technologies.

**Methods:**

A questionnaire was developed and distributed among 10 agencies known to work within this specific area. The questionnaire specifically focussed on type of sites scanned, frequency of scanning, and importance of a site for the identification of a new health technology.

**Results:**

A 100% response rate was obtained. Seven out of 10 agencies used the Internet to systematically identify new health technologies, of which 6 provided complete information. A total of 110 web sites were scanned by these 6 agencies. The number of sites scanned per agency ranged from 11 to 27. Most sites were scanned weekly (41%) or monthly (33%). Thirty-one percent (31%) of the total number of sites was considered as highly important. The agencies spent at least 2 hours a week and at most 8 hours per week scanning the Internet. Although each agency's remit differed somewhat in scope, on average the same types of sites were scanned. These include sites from regulatory agencies, sites with information on new drugs or new devices, and sites with news from newswires. However, within these types there was not much correlation between the individual sites that agencies judged important to scan.

**Conclusions:**

The use of the Internet for identifying new health technologies is increasing in the majority of horizon scanning systems around the world. At the same time there is considerable variation between individual agencies in their approach to this source of information. This can only be partially explained by differences in scope of scanning activities of the individual agencies. A coordinated effort to develop Internet search strategies for either different categories of health technologies or different clinical specialties may improve efficiency and quality of scanning in terms of the number of potentially relevant technologies identified.

## Introduction

Of the three major pressures on health services worldwide, changing demography, growing expectations of the public, and new health care interventions (technologies), the last is generating the most concern among decision makers in health care and also the most dramatic responses [1].

Health technologies are the drugs, devices, and medical and surgical procedures used in health care, and the organisational and supportive systems within which such care is provided [2]. Thus, a cardiac monitor is a technology, and an intensive care unit is also a technology.

Ideally, the introduction of new health technologies is aimed at improving the health of patients, but not all new technologies bring about health improvement or show a reasonable balance between patient benefit and costs [3]. Historical cases like DES (diethylstilbestrol) (see textbox) and many other studies have shown that new health technologies sometimes spread rapidly in a health care system, even though there is no convincing evidence on safety and effectiveness [4]. The opposite situation, although less frequent, has also been documented, resulting in underuse of beneficial and cost-effective new technologies, e.g. laser treatment of diabetic eye disease [5].

These examples illustrate that decision making on the uptake and use of a health technology needs to be supported by high quality information. An important tool for decision makers is health technology assessment (HTA). This is defined as the analysis of the implications of a health technology in terms of its safety, efficiency, effectiveness, accessibility and equity, with the aim of supporting appropriate use of health technologies by providing input to decision-making in policy and practice [6].

**The case of DES**

Diethylstilbestrol (DES) is a synthetic female hormone first produced in 1938. It has a number of uses. This case focuses on the use of DES for complications of pregnancy. DES was approved for marketing in the United States in 1941. Several uncontrolled studies carried out by the advocates of the drug presented reduced pregnancy accidents. These studies led to its frequent use in pregnancy, and were used by the industry to actively promote the use in complicated pregnancies. During the period 1950-55 there were 7 controlled studies showing DES to be ineffective. Nonetheless, promotion continued, and in the 1960's DES was frequently used over much of the world. In 1970, a rare cancer of the vagina was noted in 7 young women. In all cases, their mothers had taken DES during pregnancy. DES then gradually fell out of use in both America and Europe.

DES was a case of a treatment that was both useless and harmful. It is a reminder that technologies should be proven beneficial before they are widely used.

HTA was developed in the United States in the `70's and has since gained momentum in Europe and the rest of the world. Besides concerns on the benefits and harms of health technologies, concerns on rising expenditures for health care were an important motivation for development of the field. Increasing health care costs can partly be attributed to the growing numbers of new health technologies. To provide an illustration, during the entire decade of the 1990s, 370 new drugs were brought to market in the U.S., an increase from 239 in the 1980s. The Pharmaceutical Research and Manufacturers of America (PhRMA) states that currently, pharmaceutical and biotechnology companies have well over a thousand new medicines in clinical trials or awaiting approval by the Food and Drug Administration (FDA) [7]. This rapid change and growth has also been witnessed in other types of health technologies in recent years.

Historically, most health technology assessments focused on technologies that were in relatively widespread use in the health care setting. However, when HTA of established technologies became common practice, the need of more timely assessments was recognized. In the nineties, it was increasingly recognized that being proactive, by identifying technologies before they were licensed or launched and by producing timely assessments of these technologies, would be advantageous. In general, early notice allows decision makers time to consider possible approaches to handling a new technology within a health care system [8]. To rationalize and manage this process of early notice, Banta and Gelijns [9] stated that a systematic approach is needed to identify emerging and new technologies, to select those that are important, to assess the consequences, and finally to disseminate this information to decision makers. The systematic handling of these steps constitutes the activities of so-called Horizon Scanning Systems (HSSs). In general, the purpose of a HSS is to help control and rationalize the adoption and diffusion of new technologies in health care practice, by providing policy makers timely information on the consequences of introduction of the health technology into the health care system.

The Netherlands was among the first countries to establish a HSS [10]. Nowadays, a number of countries worldwide have established a HSS. Most horizon scanning systems evolved from the work of HTA agencies in the nineties, the main difference being the focus of HSSs on technologies early in their life cycle. As a consequence, many HSSs are part of, or connected to HTA agencies.

The majority of these systems are members of The European Information Network on New and Changing Health Technologies, EuroScan , representing agencies in Canada, Denmark, France, Israel, Norway, Spain, Sweden, Switzerland, The Netherlands, and The United Kingdom [11]. All HSS in these countries are at least 50% funded from public sources, and target central and local health care policy makers with their early assessments. Furthermore, there are a number of organisations that provide the same services and use the same methods, but are not government funded. These include the Australian Safety and Efficacy Register of New Interventional Procedures- Surgical (ASERNIP-S) in Australia, and the University HealthSystem Consortium (UHC) in the United States.

In general, HSSs are interested in identifying potentially significant technologies for health and health care that might become available on the market in 0 to 5 years time. To identify these technologies, various sources have been recommended including consultation of individual manufacturers and clinicians, written sources such as pharmaceutical and medical journals, and the World Wide Web [1]. A combination of sources is recommended, as this provides corroboration, increases the likely accuracy of any predictions and increases the amount of useful information regarding a new technology [12].

Of the different types of sources, the Internet holds the promise of timely and efficient searching. However, although HSSs have begun to use the Internet as a source of information, anecdotal evidence suggests that its use varies and is generally unsystematic. As the efficient identification of new health care technologies is of the utmost importance for HSSs, this paper describes and analyses the current use of the Internet for this purpose by members of EuroScan and other selected horizon scanning agencies.

## Methods

Drawing on a previous survey carried out internally within EuroScan, those horizon scanning agencies who use the Internet in one way or another in the operation of their system were identified. A questionnaire was sent to these agencies (n= 8) in April 2002. In addition, it was sent to two agencies outside the network that identify and assess emerging health technologies.

The questionnaire included 10 questions covering the following topics:

- The scope of the scanning activity
- The sources used and strategy employed for identification of new health technologies
- The amount of time available to scan

The first questions addressed what type of technologies the agencies scanned for, and which clinical specialties were priority areas for the scanning activity. Furthermore, agencies were asked which web sites they visited and were asked to appraise the selected sites to establish which sites were the most useful for them. The reason for these questions was to see if the agencies employed a strategy for which sites to scan based on type, importance of the site and the frequency of scanning. Finally, the agencies were consulted on how in general they prioritise web sites, and how much time they had available per week for scanning.

## Results

All 10 agencies responded (100% response rate). At the time of the survey, three agencies (30%) indicated that they did not use the Internet to systematically identify new health technologies. These are the Health Council in the Netherlands, the Committee for Evaluation and Diffusion of Innovative Technologies (CEDIT) in France, and the Agencia de Evaluacion de Tecnologias Sanitarias (AETS) in Spain. In these agencies the Internet was used as a secondary source that is, to search for information on already identified technologies to enable them to prioritise these. The remaining agencies (n=7) all used the Internet as a primary source of information (see Table 1). Six of these completed the questionnaire.

**Table 1:** Countries and organizations operating Horizon Scanning Systems using the Internet to identify new health technologies

**HSS**	**Acronym**	**Country**
Australian Safety and Efficacy Register of New Interventional Procedures-Surgical	ASERNIP-S	Australia
Canadian Emerging Technology Assessment Program at the Canadian Coordinating Office for Health Technology Assessment (CCOHTA)	CETAP	Canada
Danish Centre for Evaluation and Health Technology Assessment	DACEHTA	Denmark
Programa de Evaluación de Tecnologi´as Emergentes at the Servicio de Evaluación de Tecnologi´as Sanitarias (OSTEBA)	SorTek	Spain (Basque country)
Federal Social Insurance Office of Switzerland	FSIOS	Switzerland
National Horizon Scanning Centre	NHSC	United Kingdom
University HealthSystem Consortium	UHC	United States of America

### Scope of scanning

Table 2 summarizes the responses of the agencies on which type of technology they look for (drugs or non-drugs), which specialty areas are included in the scanning activity, and if they scan different types of sites to identify different types of technology.

Table 2 shows that most agencies scan for all types of health technologies. Two agencies, ASERNIP-S and SORTEK limit their scanning activity to identify new medical devices and procedures. In addition, all agencies, except ASERNIP-S and DACEHTA, focus their scanning activities on all specialty areas. These latter agencies focus on surgery and oncology, respectively. The final row in the table indicates if agencies scan different types of sites to identify drugs or medical devices and procedures. Four agencies say they do not, and two agencies say they do scan different sites for different types of technologies.

**Table 2:** Scope of scanning of Horizon Scanning Systems using the Internet to identify new health technologies

**Scope of scanning**	**Agencies**						
	CETAP	NHSC	DACEHTA	SORTEK	FSIOS	ASERNIP-S	UHC
*Type of technology*	Medical devices and procedures, and drugs	Medical devices and procedures, and drugs	Medical devices and procedures, and drugs	Medical devices and procedures	Medical devices and procedures, and drugs	Medical devices and procedures	Medical devices and procedures, and drugs
*Specialty areas*	All	All	Oncology	All	All	Surgery	All
*Different sites for different types of technologies*	No	No	No	Yes	n.a	No	Yes

### Scanning strategy: frequency, relative importance of sites, and types of sites

Of the seven agencies that use the Internet as a primary source of information to identify new health technologies, six provided information on the frequency of scanning, the URL of sites scanned, and their relative importance. In total, the agencies scanned 110 different web sites. One agency, University HealthSystem Consortium, provided us with a list of 52 sites scanned, which have been described as part of a chapter in an electronic textbook on resources for Health Technology Assessment. This textbook is located at the web site of the National Library of Medicine [13].

The total number of sites scanned by the 6 agencies that provided us with complete information ranged from 11 to 27. The frequency of scanning is shown in Table 3.

**Table 3:** Frequency of scanning of web sites by 6 Horizon Scanning Systems

**Frequency**	**No. of web sites (%)**
**Daily**	19 (17)
**Weekly**	45 (41)
**Bi-weekly**	8 (7)
**Monthly**	36 (33)
**Listservs**	2 (2)
**Total**	110 (100)

Table 3 illustrates that most of the web sites are scanned weekly (41%) or monthly (33%). Two web sites provided listservs, that is, a service that sends selected information from the site to your personal e-mail address on a daily or weekly basis. For each agency, the number of web sites scanned daily ranged from 1 to 5 web sites, and the number scanned weekly ranged from 3 to 15 web sites.

Thirty-one percent of the total number of websites is considered highly important for the identification of new health technologies. Forty-three percent of sites were considered important, and 24% less important. Two sites were not evaluated, as there had been limited experience of scanning these sites (see Table 4).

**Table 4:** Importance of scanned web sites as evaluated by 6 Horizon Scanning Systems

**Evaluation**	**No. of web sites (%)**
**Highly important**	34 (31)
**Important**	47 (43)
**Less important**	27 (24)
**Not appraised**	2 (2)
**Total**	110 (100)

### Importance of sites

Six agencies provided information on the relative importance of individual web sites in their selection of routinely scanned sites. The web sites that were judged `highly important', `important' and `less important' for the identification of new health technologies are listed in Table 5,Table 6, and Table 7 respectively. Out of a total of 110 sites judged, 16 (15%) sites were evaluated by more than one agency.

**Table 5:** Highly important web sites for identifying new health technologies as evaluated by 6 Horizon Scanning Systems

**Web sites**		
**Type of information**	**Source of information**	**Location/address of web site (URL)**
***Regulatory information***	The European Agency for the Evaluation of Medicinal Products (EMEA)	http://www.emea.eu.int/
	Food and Drug Administration (FDA)FDA-NEWSDIGEST-L (listservs)	http://www.fda.gov/emaillist.html
	F-D-C Reports	http://www.fdcreports.com/
	FDA Oncology Tools	http://www.fda.gov/cder/cancer/index.htm
***Information on new drugs***	DrugInfoZone	http://www.druginfozone.org/news/news.htm
	PharmaLive	http://www.pharmabusiness.com/
***Information on new medical devices***	Medical Data International	http://www.medicaldata.com/
***Developments in science***	NewScientist (online journal)	http://www.newscientist.com/
	Science Daily Magazine	http://www.sciencedaily.com/index.htm
***Newswires***	Reuters Health	http://www.reutershealth.com/frame2/eline.html
	Ivanhoe Medical Breakthrough	http://www.ivanhoe.com/home/p_home.cfm
***Specialty-specific sites (surgery and oncology)***	SurgeryLinx	http://www.surgerylinx.com/
	American Cancer Society	http://www.cancer.org/docroot/nws/nws_6.asp?level=1
	Cancersource	http://www.cancersourcemd.com/news/index.cfm
	Doctor's Guide	http://www.pslgroup.com/dg/haematonews.htm
	Oncology Week in Review	http://www.cancereducation.com/CancerSysPages/OWR/listarticles.cfm?cncr=49
	Medscape Hematology-Oncology	http://www.medscape.com/hematology-oncologyhome?pagename=oncology
***Other Horizon Scanning or HTA organisations***	National Horizon Scanning Centre	http://www.publichealth.bham.ac.uk/horizon
	Australian Safety and Efficacy Register-Surgery	http://www.surgeons.org/asernip-s/
	Canadian Coordinating Office for HTA	http://www.ccohta.ca/
	Swedish Early Warning System - SBU ALERT	http://www.sbu.se/admin./index.asp
	The European Information Networkon New and Changing Health Technologies (EuroScan)	http://www.publichealth.bham.ac.uk/euroscan/
	Alberta Heritage Foundation for Medical Research (AHFMR )	http://www.ahfmr.ca/
	Succinct and Timely Evaluated Evidence Review (STEER)	http://www.soton.ac.uk/~wi/projx/signpost/welcome1.htm

The sites have been categorised into different types, according to their main features and purpose, such as sites containing regulatory information on drugs and devices for the US and Europe, information on specific types of technologies (drugs, devices, procedures), information on specific specialties, information from newswires, and information on new health technologies identified and/or evaluated by other agencies (see Table 5).

Of the sites containing regulatory information, the FDA (United States Food and Drug Administration) web sites are scanned by both European and North American agencies and are rated as highly important. The FDA provides a free e-mail service for news on both newly approved drugs and medical devices, FDA-NewsDigest-L (the Center for Devices and Radiological Health for devices, and the Centre for Drug Evaluation and Research for drugs), which users rated as highly important. In addition, the European Agency for the Evaluation of Medicinal Products (EMEA) web site provides information on approved drugs in the European Union, and outlines the evidence base for approval. On a commercial basis, the F-D-C reports' site allows access to the table of contents and brief summaries of information contained in F-D-C publications. Of these, the `Pink Sheet' covers the latest regulatory, legislative and business news affecting the US prescription pharmaceutical industry (http://www.thepinksheet.com/FDC/Weekly/pink/TOC.htm). The `Gray Sheet' focuses on medical devices, diagnostics and instrumentation (http://www.fdcreports.com/grayout.shtml).

**Table 6:** Important sites for identifying new health technologies as evaluated by 6 Horizon Scanning Systems

**Web sites**		
**Type of information**	**Source of information**	**Location/address of web site (URL)**
**Regulatory information**	Medical Devices Agency	http://www.medical-devices.gov.uk/
**Information on new medical devices**	Medical Device Daily	http://www.medicaldevicedaily.com/
	Biomednet	http://news.bmn.com/latest
	Medical Design Online news	http://www.medicaldesignonline.com/
**Health portals**	Doctor's Guide	http://www.docguide.com/
	Medscape	http://www.medscape.com/
	EurekAlert	http://www.eurekalert.org/
	Doctorinfoline	http://www.doctorinfoline.com/
	National Electronic Library for Health	http://www.nelh.nhs.uk/hth/archive.asp
**Developments in science**	Science Daily Magazine	http://www.sciencedaily.com/index.htm
**Newswires**	CNN.com Health	http://www.cnn.com/HEALTH
	New York Times on the Web health section	http://www.nytimes.com/pages/health/index.html
	UK health news digest (from BMJ)	http://bmj.com/uknews/
	Future Health Bulletin	http://www.headstar.com/futurehealth/subs.html
**Specialty-specific sites (surgery)**	Foxhall Surgery	http://www.foxhall.com/

One of the agencies, the University HealthSystems Consortium (UHC), further recommends the NDA pipeline compiled by F-D-C reports, a weekly updated database for tracking drug and biological product research, clinical trials and approvals. It is accessible on subscription basis only (http://www.ndapipeline.com/c3/welcome/welcome.plex).

For news on drugs in the pipeline, DrugInfoZone was used by two agencies. Most information on this site is password protected (for National Health Service staff in the UK only). The site includes, amongst others, a database of recent drug launches, a patents database, and drug reviews. Furthermore, it provides a free daily e-mail service with news from a number of sources including newslines such as Reuter's health, medical journals, pharmaceutical journals and other health-related web sites. The PharmaLive site was scanned by one agency. It is a web site targeted towards the pharmaceutical industry, providing news items on research, marketing, and regulation of drugs. It is a commercial web site. Access to more detailed pipeline information is on paid subscription basis only. The only web site rated as highly important that Horizontal Scanning Systems agencies identified as providing valuable information about medical devices is Medical Data International. However, this news service has recently introduced subscription fees. Reuters Health's web site is recommended for news on new health technologies in general. This web site is used as a primary source by many other health information web sites as well. Examples of specialty-specific web sites are SurgeryLinx, providing summaries and access to journal articles on new surgical procedures, and Doctor's Guide Haematonews, which lists news items on all types of cancer.

Table 6 shows the sites that were valued as important by the responding agencies.

Table 6 shows that, compared with Table 5, a new category of sites `Health portals' has come up. Health portals includes sites like Medscape and Doctor's Guide to the Net, that report on information on different types of health technologies from newswires, clinical conferences, and journals, and provide the possibility to search for this information in a great variety of predefined clinical specialties. The categories `Information on new drugs' and `Other EW or HTA organisations' have disappeared, because no sites were listed in these categories.

Table 7 shows the sites that were valued as `less important' by the agencies.

Compared to Table 5 and Table 6,Table 7 shows two new types of sites: `Consumer Health Information' and `Journals'. The category of `Information on new drugs' does not exist in this listing of sites.

**Table 7:** Less important web sites for identifying new health technologies as evaluated by 6 Horizon Scanning Systems

**Web sites**		
**Type of information**	**Source of information**	**Location/address of web site (URL)**
**Regulatory information**	Food and Drug Administration (FDA)	http://www.fda.gov/
**Information on new medical devices**	MedicalDesignOnline	http://www.medicaldesignonline.com/content/homepage/default.asp
	BiomedNet	http://news.bmn.com/latest
	MedBizPeople	http://www.medbizpeople.com/news/XcNewsPlus.asp?cmd=LIST
**Health portal**	EurekAlert	http://www.eurekalert.org/
	Medynet	http://www.medynet.com/
	National ElectronicLibrary for Health	http://www.nelh.nhs.uk/hth/archive.asp
**Developments in science**	Beyond 2000	http://www.beyond2000.com/news/medicine.html
**Newswires**	BBC	http://news.bbc.co.uk/
	ABC Health	http://www.abc.net.au/health/
	Reuters Health	http://www.reutershealth.com/
	BBC science	http://www.bbc.co.uk/science/tw/2002/
	Yahoo News (cancer)	http://story.news.yahoo.com/fc?cid=34&tmpl=fc&in=Health&cat=Cancer_Research
	PR newswire	http://www.prnewswire.co.uk/newsindex.shtml
	Financial Times (subscription)	http://www.ft.com/
	American Medical News	http://www.amednews.com/
**Specialty-specific site (cardiology)**	About.com	http://heartdisease.about.com/cs/newtechniques/
**Consumer health information**	InteliHealth	http://www.intelihealth.com/IH/ihtIH/WSIHW000/408/408.html
**Journals**	Journal of the American Medical Association (JAMA)	http://jama.ama-assn.org/
	Archives of Surgery	http://archsurg.ama-assn.org/
**Other Horizon Scanning or HTA organisations**	Hayes Inc.	http://www.hayesinc.com/productsandservices_medicaltechnologydirectory.htm

### Overlap in sites scanned

Table 8 presents an overview of the 16 sites that were scanned by more than one agency.

Table 8 shows that half of the sites are only scanned by 2 agencies. In addition, in the vast majority of sites there is a discrepancy between the agencies with regard to their relative importance. For example, Medscape is scanned by 5 agencies, and is valued as highly important by one agency (indicated by 1), and as important site by the other four (indicated by 2). Medscape is a health portal, and provides information from newswires, journals, and conferences on a variety of clinical specialties. This information can be accessed on their home page, but also by specialty on specialty pages, for example Medscape Haematology-Oncology.

Reuters Health is scanned by 4 agencies. This site is valued very differently, from highly important by one agency, important by another and less important by a further 2 agencies. In general, Reuters Health is considered a valuable source for news related to health and medicine. The site provides abstracts on news items that enable users to judge the item's value.

The EuroScan web site is valued as either highly important or important by the four agencies that scan this site. The site is mostly visited by members of this information network. The site contains a database, only accessible to member agencies, which includes information from members on new health technologies, enabling exchange of information between the members.

**Table 8:** Overlap in sites scanned between agencies and differences in evaluation of individual web sites

**Web sites**		**Number of agencies scanning this site**	**Appraisal**
***Health portals***	Medscape http://www.medscape.com	5	1,2,2,2,2
***Newswires***	Reuters Health http://www.reutershealth.com	4	1,2,3,3
***Other EW or HTA organisations***	The European Information Networkon New and Changing Health Technologies (EuroScan) http://www.publichealth.bham.ac.uk/euroscan	4	1,1,2,2
***Health portals***	Doctor's Guide to the Internet http://www.docguide.com	3	1,2,2
	EurekAlert http://www.eurekalert.org	3	2,2,3
	Ivanhoe Medical Breakthroughs http://www.ivanhoe.com	3	1,2,2
***Other horizon scanning or HTA organisations***	Swedish Early Warning System - SBU ALERT http://www.sbu.se/admin./index.asp	3	1,1,2
	Australian Safety and Efficacy Register-Surgery http://www.surgeons.org/asernip/	3	1,1,1
***Regulatory information***	FDA Oncology Tools http://www.fda.gov/cder/cancer/	2	1,2
	F-D-C reports http://www.fdcreports.com/	2	1,3
***Information on new drugs***	DrugInfoZone http://www.druginfozone.org/news/news.htm	2	1,1
***Information on new medical devices***	Medical Design Online http://www.medicaldesignonline.com	2	2,3
	BioMedNet news http:/news.bmn.com/latest	2	2,3
	Medical Data International http://www.medicaldata.com/	2	1,2
***Newswires***	American Medical News http://www.amednews.com	2	3,3
***Other Horizon Scanning or HTA organisations***	Canadian Coordinating Office for HTA http://www.ccohta.ca	2	1,1

### Identification and prioritisation of new web sites

The responses to this question indicate that new web sites are mostly found through word of mouth (colleagues), or through links from one site to another site. Frequently, new sites are prioritised by an information specialist in an informal way. One agency's response was that sites are trialled for 1-2 months and that after that a recommendation is made to include or exclude the site in the routine scanning activity. In either method of prioritising sites, the same set of criteria is used to prioritise one site above another. The agencies responded that sites that appear to produce more or a similar amount of useful information than sites that are already scanned are likely to be added to the list of sites to scan. Furthermore, sites are most attractive when they are easy to scan, provide an e-mail service, are free of charge, and when they appear to provide objective information.

### Available time to scan web sites

The responding agencies use at least 2 hours, and at most 8 hours per week to scan. Of the agencies, one scans less than 3 hours, 4 scan between 3-6 hours a week, and another up to 8 hours a week. One agency provided a range of 2-8 hours.

## Discussion

Although the absolute number of agencies that have been covered by the survey is small, we have reasons to believe that it has covered most, if not all, HSS in the industrialized world. . Firstly, through cooperation with EuroScan it was possible to identify all member agencies that in one way or another use the Internet as part of their horizon scanning system. These agencies were complemented by two other agencies that carry out horizon scanning activities but who were not members of EuroScan. Because of the nature of the horizon scanning activity the vast majority of agencies in our sample is publicly funded, mostly by central governments. Agencies also tend to be relatively small, consisting of 1.5 to 7 full-time equivalents [14]. In addition, scanning is only one of the activities involved in the operation of most HSSs, with prioritisation and early assessments of new health technologies being other important functions. As a consequence of this resources for scanning are limited. Taking into consideration that the number of health technologies that emerge from pharmaceutical pipelines and manufacturers portfolios is great, the need for an efficient scanning strategy for EW agencies is evident.

The general picture that emerges from the results is that around half of the horizon scanning agencies actively uses the Internet as a source of information for identifying new health technologies. Furthermore, the agencies that have made this step on average spend considerable time on this activity. This is illustrated by the fact that the majority of the agencies scan for 3-6 hours a week, and that around 40% of selected sites are scanned weekly with some being scanned daily.

In total, the agencies scan a large number of sites (n=110). However, only 15% of these sites are scanned routinely by more than one agency. The relative lack of overlap in sites scanned can be partly explained by differences in scope of scanning between different agencies (e.g. only focusing on drugs versus including all technologies). Other factors that contribute to the diversity of sites being scanned and the lack of overlap of scanning activities between agencies include the great number of web sites available on the Internet, and the fact that sites are frequently selected in an unsystematic, informal way. Individual preferences of local information specialists may therefore be of paramount importance for the outcome of the selection process.

Similarly, differences between agencies in their rating of individual sites may occur due to the factors listed above. In this regard, the finding that 24% of the sites scanned were judged as less important has been surprising. One may wonder why agencies scan less important sites, when at the same time resources are limited? One explanation could be in what Wagner [13] defines as a preferred method of scanning, including the scanning of both `core' and `adjunct' sources (sites), that is first scanning those sites that have proved to yield most valuable information, and when there is time left, scanning additional sites that could yield supplementary information.

We feel that this subjective assessment by the agencies, of the relative importance of web sites that they scan, could serve as a starting point for discussion between agencies in order to arrive at common criteria to determine the usefulness and importance of web sites for identifying new and emerging health technologies.

Although a large number of sites are scanned (n=110), these can be categorised into a much smaller number of types of sites (n=10) that have a similar purpose. The categorization has been made on the basis of the main feature and purpose of a web site, and is as such not totally mutually exclusive. Of these categories, some are used more and are rated higher than others. Prominent types are `Regulatory information', `Information on new drugs', `Specialty-specific sites', `Newswires', sites of `Other Horizon Scanning or Health Technology Assessment organisations', and possibly `Health Portals'. This might point to their importance to include in a search strategy for identification of new health technologies.

We conclude from the survey that there is marked variation between horizon scanning agencies in the way they use the Internet for identifying new health technologies. We have the impression that these differences can only partly be explained by differences between individual agencies in terms of e.g. source of funding, scope of scanning, and so forth. Factors that may be equally or even more important in explaining variation are that identification of technologies using the Internet is a rather new activity, and that so far there has only been limited exchange of information on this subject between agencies. We therefore recommend, given the resources used on scanning, that agencies become more selective in their choice of web sites and perhaps try to define a more transparent, operational distinction between highly important, important, and less important sites for the identification of new health technologies.

Horizon scanning agencies may benefit from further investigation into which sites deliver most output. Exchange of information between agencies about valuable sites, and a more formal selection process of new web sites on the basis of selected criteria could result in a more efficient scanning process. A future activity could include a coordinated effort to develop Internet scanning strategies for different categories of health technologies or different clinical specialties. This may improve efficiency and quality of scanning in terms of numbers of potentially relevant technologies identified.

In practice, the Internet does not stand alone as a source. Most agencies use a combination of sources, such as information from clinical experts and manufacturers, scientific journals, grey literature, and conference material. It is therefore furthermore recommended that future Internet scanning strategies fit into the broader search strategy of agencies.
